# Isolation and genomic analysis of circulating tumor cells from castration resistant metastatic prostate cancer

**DOI:** 10.1186/1471-2407-12-78

**Published:** 2012-02-28

**Authors:** Mark Jesus M Magbanua, Eduardo V Sosa, Janet H Scott, Jeff Simko, Colin Collins, Dan Pinkel, Charles J Ryan, John W Park

**Affiliations:** 1 Department of Medicine, Division of Hematology/Oncology, University of California San Francisco (UCSF), San Francisco, CA, USA; 2 Department of Pathology and Urology, UCSF, San Francisco, CA, USA; 3 Vancouver Prostate Center and Department of Urologic Sciences, University of British Columbia, Vancouver, BC, Canada; 4 Department of Laboratory Medicine, UCSF, San Francisco, CA, USA; 5 Helen Diller Family Comprehensive Cancer Center, UCSF, San Francisco, CA, USA; 6 Division of Hematology/Oncology, University of California San Francisco, 2340 Sutter St. Box 1387, San Francisco, CA 94115, USA

**Keywords:** Circulating tumor cells, Castration resistant prostate cancer, Copy number analysis, Array comparative genomic hybridization, Androgen receptor

## Abstract

**Background:**

The number of circulating tumor cells (CTCs) in metastatic prostate cancer patients provides prognostic and predictive information. However, it is the molecular characterization of CTCs that offers insight into the biology of these tumor cells in the context of personalized treatment.

**Methods:**

We developed a novel approach to isolate CTCs away from hematopoietic cells with high purity, enabling genomic analysis of these cells. The isolation protocol involves immunomagnetic enrichment followed by fluorescence activated cell sorting (IE/FACS). To evaluate the feasibility of isolation of CTCs by IE/FACS and downstream genomic profiling, we conducted a pilot study in patients with metastatic castration resistant prostate cancer (CRPC). Twenty (20) sequential CRPC patients were assayed using CellSearch™. Twelve (12) patients positive for CTCs were subjected to immunomagnetic enrichment and fluorescence activated cell sorting (IE/FACS) to isolate CTCs. Genomic DNA of CTCs was subjected to whole genome amplification (WGA) followed by gene copy number analysis via array comparative genomic hybridization (aCGH).

**Results:**

CTCs from nine (9) patients successfully profiled were observed to have multiple copy number aberrations including those previously reported in primary prostate tumors such as gains in 8q and losses in 8p. High-level copy number gains at the androgen receptor (*AR*) locus were observed in 7 (78%) cases. Comparison of genomic profiles between CTCs and archival primary tumors from the same patients revealed common lineage. However, high-level copy number gains in the *AR *locus were observed in CTCs, but not in the matched archival primary tumors.

**Conclusions:**

We developed a new approach to isolate prostate CTCs without significant leukocyte admixture, and to subject them to genome-wide copy number analysis. Our assay may be utilized to explore genomic events involved in cancer progression, e.g. development of castration resistance and to monitor therapeutic efficacy of targeted therapies in clinical trials in a relatively non-invasive manner.

## Background

Cancer metastasis typically occurs through hematogenous spread, and thus strategies to detect tumor cells in the blood have been developed. Such detection methods may be used in applications such as staging/prognosis, disease monitoring, and studies of the metastatic process [1-3]. For example, monitoring of CTC levels via immunomagnetic enrichment and fluorescence based microscopy (Cell Search™, Veridex LLC) has been shown to be prognostic in metastatic breast cancer patients and predictive of treatment response [4,5]. In metastatic prostate cancer, de Bono et al [6] demonstrated that CTC levels as enumerated by this approach predicted overall survival better than PSA decrement algorithms in the CRPC patient population. The presence of ≥ 5 CTCs per 7.5 mL of blood at any time point was significantly associated with poor prognosis.

The biological significance of CTCs remains unknown, underscoring the need for functional analysis and molecular characterization of these cells. Furthermore, the role of CTC detection in the clinical management of prostate cancer remains debated. For example, the relation of CTCs to currently used diagnostic tools, such as clinical nomograms, is unclear. Therefore, it is of interest to develop approaches to further characterize these cells. Detailed molecular characterization of CTCs, however, has been hampered because of their rarity and the difficulty of isolating CTCs for analytical techniques [for review see Ref. [3]].

Here we describe a new approach for prostate CTC isolation and genomic profiling, using immunomagnetic enrichment (IE) and fluorescence activated cell sorting (FACS) to purify CTCs, and array comparative genomic hybridization (aCGH) to evaluate copy number aberrations. We evaluated our assay in a pilot study involving patients with CRPC who had received chemotherapy. Using this protocol, CTCs were isolated and profiled for copy number aberrations via aCGH analysis following whole genome amplification (WGA). Results of copy number analysis confirmed their malignant nature and revealed aberrations commonly seen in primary prostate cancer. Additionally, our results suggested that CTCs isolated via IE/FACS were highly pure without significant dilution by hematopoietic cells.

## Methods

### Patient samples

Clinical samples were obtained from 20 castration resistant prostate cancer patients who were recruited from November 2008 to August 2009 at the University of California San Francisco (UCSF) to participate in our study (Table 1). Twenty patients were selected based on statistical considerations for a pilot study. All patients gave informed consent under a protocol approved by the UCSF Institutional Review Board. Blood was collected in three CellSave™ tubes (Veridex). This allowed us to use one tube for an initial CTC enumeration using the published CellSearch™ method, and the two remaining tubes to be combined for IE/FACS isolation. Microdissection and DNA extraction of archived formalin-fixed paraffin-embedded (FFPE) tumor biopsy samples from two patients were done in a manner as previously described [7]. Using H&E stained slide, the pathologist (J.S.) demarcated tissue containing at least 80% tumor. The H&E stained slide was then used as a guide to microdissect the tumor area using a surgical blade (Feather #15) on non-stained deparaffinized sections (10 sections × 5 μM thickness). In the case of a small tumor (Patient #20), manual microdissection of the selected area was performed by scraping successive tissue sections under a stereomicroscope.

**Table 1:** Enumeration and genomic profiling of circulating tumor cells (CTCs) from 20 castration resistant prostate cancer patients

**Patient No**.	CTC/7.5 mL	IE/FACS	Days from draw to IE/FACS	No. of cells collected as input for WGA	aCGH
1	1	n.d.	N.A.	N.A.	N.A.
2	1	n.d.	N.A.	N.A.	N.A.
3	85	Yes	1	50	Yes
4	12	Insufficient sample	N.A.	N.A.	N.A.
5	144	Failed due to machine error	1	N.A.	N.A.
6	6	Insufficient sample	N.A.	N.A.	N.A.
7	10	Insufficient sample	N.A.	N.A.	N.A.
8	2	n.d.	N.A.	N.A.	N.A.
9	590	Yes	1	20 × 2	Yes (duplicate)
10	151	Yes	4	20	Yes
11	9	Yes	2	20	Yes
12	2	n.d.	N.A.	N.A.	N.A.
13	55	Yes	1	20	Yes
14	590	Yes	2	20	Yes
15	42	Yes	2	33	WGA failed
16	0	n.d.	N.A.	N.A.	N.A.
17*	54	Yes	1	20	Yes
18	33	Yes	1	20	Yes
19	34	Yes	2	20	Hybridization failed
20*	12	Yes	1	18	Yes

*Patients with available matched primary tumor

IE/FACS-immunomagnetic enrichment and fluorescence activated cell sorting; aCGH-array comparative genomic hybridization; WGA-whole genome amplification; n.d.-not done; N.A.-not applicable

### Patient eligibility criteria

This pilot study was conducted among men with prostate cancer who met the following criteria: histologically-proven castration resistant prostate cancer that has metastasized; prior therapy with ≥ 3 cycles (9 weeks) of docetaxel based chemotherapy; progressive disease within the last 1-2 months as characterized by rising PSA level, new lesions on radionuclide bone scan or computed tomography scans; last dose of docetaxel ≥ 21 days prior to blood collection; no chemotherapy within the previous 21 days and no radiation therapy within 21 days.

### Enumeration of CTCs

To increase the likelihood of isolating CTCs for molecular analysis, we first performed CTC enumeration using the automated CellSearch™ assay to identify patients with > 5 CTCs per 7.5 mL. We then isolated CTCs by IE/FACS (see below) from the remaining blood sample in these patients. Cell counts via CellSearch™ correlated closely with cell counts via IE/FACS (Park, unpublished data). In initial studies, we observed that whole genome amplification of DNA from CTCs was less efficient when CTCs were isolated after 48 h post-phlebotomy. Therefore, enumeration was performed within 24 h and isolation of CTCs via IE/FACS within 48 h after blood draw.

### Cell isolation via IE/FACS

For immunomagnetic enrichment, one-half volume of Cell Buffer (Immunicon) was added to a blood sample. Immunomagnetic particles coated with EpCAM (MJ37) mAb (50 μg/10 mL of indirect conjugate or 60 μg/10 mL for the direct conjugate) and EpCAM (EBA-1) mAb conjugated to phycoerythrin (PE) (400 μL at 5 μg/mL per 10 mL of sample) were added to the sample. The sample was incubated at room temperature for 15 min, mixing at half-way point and again when incubation was completed. The sample was placed in a magnetic separator (Immunicon) at room temp for 15 min, and then aspirated to remove unbound blood cells. The bound cells were eluted in 2 mL of Cell Buffer, moved to a 12 × 75 mm tube and subjected to a second round of magnetic separation for 5 min. The fluid in the tube was aspirated and the remaining cells were resuspended in 150 μL of Cell Buffer. Twenty μL of a solution containing 2 μg of a proprietary (BD Bioscience) nucleic acid dye, and 0.1 μg of a leukocyte-specific CD45 (2D1) mAb conjugated to peridinin-chlorophyll-protein-Cy5.5 were added to resuspended cells. The sample was incubated in the dark for 15 min and then sorted using FACS Aria (BD Biosciences). CTCs were defined as nucleated, EpCAM-positive, and CD45-negative while leukocytes were defined as CD45-positive, EpCAM-negative and nucleated. Cells were sorted into 10 μL of TE (10 mM Tris-HCl 1 mM EDTA pH 8.0) in a 500 μL PCR tube. All conjugated antibodies and reagents for FACS were obtained from BD Biosciences unless otherwise noted. During FACS analysis, we sorted a range of 18-50 CTCs. Table 1 shows the number of "input cells" used for WGA, and the number of replicate analyses performed for each subject. Due to the small numbers of CTCs isolated from patients' blood, it was not feasible to perform FACS analysis after sorting. Also, since blood samples were collected in CellSave™ (Veridex) tubes [6,8], CTCs were thereby rendered nonviable and fixed to maintain antigenicity prior to immunomagnetic enrichment and FACS sorting. Complete FACS plots from two representative patients are shown in Additional file 1: Figure S1.

### Optimization of whole genome amplification (WGA)

ACGH protocols typically use between 100 ng and 1 μg of specimen DNA in the labeling reaction, equivalent to ~20,000-200,000 cells, which does not permit reliable analysis of important samples containing small numbers of tumor cells such as CTCs in blood. Analyzing these samples for copy number aberration requires a high-fidelity whole genome amplification (WGA) step. To select the best strategy for WGA of limiting amounts of input DNA, we tested four WGA protocols: multiple displacement amplification [9], random prime amplification [10], ligation-mediated-PCR [11], and the GenomePlex^® ^WGA4 [12,13]. We compared aCGH results generated from diluted BT474 breast cancer cell line genomic DNA (equivalent to ~10,000 to 10 cells) to non-limiting unamplifed BT474 DNA (600 ng). We found GenomePlex^® ^WGA4 to be most reliable and reproducible (data not shown). We also did not observe artifacts or bias via BAC aCGH analysis. Other groups have also demonstrated the reproducibility and reliability of the GenomePlex^® ^WGA protocol [12,14].

### Whole genome amplification (WGA)

To prevent any loss of genomic material, we avoided the use of column purification of genomic DNA. Instead, whole cell lysis of isolated cells and WGA of genomic DNA were performed in the same PCR tube the cells were sorted into. Genomic DNA in the whole cell lysate served as template for WGA which was performed using the GenomePlex^® ^Single Cell WGA4 Kit (Sigma-Aldrich) following the manufacturer's protocol. Briefly, 1 μL lysis and proteinase K digestion solution was added to cells in 10 μL of TE. The DNA was then fragmented and libraries were prepared. Amplification was performed by adding 7.5 μL of 10 × Amplification Master Mix, 48.5 μL of nuclease-free water and 5 μL WGA DNA polymerase. Samples were amplified using an initial denaturation of 95°C for 3 min followed by 25 cycles, each consisting of a denaturation step at 94°C for 30s and an annealing/extension step at 65°C for 5 min. Amplified DNA was purified using the QIAQuick PCR Purification Kit (Qiagen). DNA concentration was determined by a NanoDrop™ spectrophotometer and stored at -20°C. The average yield after WGA of ~20 CTCs is ~10 μg. Early tests via aCGH analysis of samples with WGA product yield less than < 5 μg revealed non-specific amplification. Therefore samples with low yield (< 5 μg) were excluded from further downstream assays.

### Array CGH analysis

In initial studies we performed aCGH analysis using oligonucleotide microarrays (Affymetrix 50 K SNP array), but observed significant experimental variability and background noise with our WGA4 amplified DNA samples. Similarly, studies by Fuhrmann et al [15] and Nowak et al [14] showed that bacterial artificial chromosome (BAC) arrays outperformed oligonucleotide microarrays in detecting small gains or losses [15] and showed far fewer outliers and less technical noise [14]. So, we performed our aCGH analysis using a bacterial artificial chromosome (BAC) array (Version 3.2) containing 2,464 clones printed in triplicate [16]. The BAC arrays are available at the Helen Diller Family Comprehensive Cancer Center Array Core.

The random prime amplification method [16] was used to enzymatically label approximately 600 ng of test DNA (WGA product) or unamplified normal female genomic (Promega) reference DNA with Cyanine 3-dCTP or Cyanine 5-dCTP (Amersham), respectively. Labeled test and reference DNA were co-hybridized to a BAC array. The arrays were imaged using a custom built CCD array imaging system [17] and analyzed using the UCSF Spot Program to calculate Cy3/Cy5 ratios. The UCSF Spot Program is a fully automated system for microarray imaging that locates both subarray grids and individual spots [18]. Ratios are then computed based on explicit segmentation of each spot. The resultant log2 ratios were analyzed by UCSF Sproc to yield the mean and standard deviation of the log2 ratios over replicate spots. The UCSF Sproc is a companion program to UCSF Spot that maps each spot on the array to a specific clone and chromosome position, and averages over replicate spots in order to output a final ratio value for each clone on the array. Clones with a standard deviation of the log2 ratios of the triplicate spots greater than 0.2 for were excluded from analysis.

To determine copy number alterations, microarray data (sproc files) was uploaded and analyzed using Nexus 5.1 software (Biodiscovery). Data was subjected to rank segmentation, a proprietary (Biodiscovery) variation of the circular binary segmentation (CBS) [19]. The thresholds of log2 ratio values for gains and losses were 0.2 and -0.2 respectively; the thresholds for high copy number gains and homozygous deletions were 0.6 and -0.6 respectively. Copy number gains or losses were considered recurrent if present in > 50% of the CTC samples from 9 patients.

Since sex-mismatch hybridization was performed, i.e. male test (tumor) versus female (normal) reference, we analyzed copy number alterations on chromosome X by using a feature available in the Nexus 5.1 software for automatic threshold adjustment on the X chromosome. Theoretically, the intensity of a normal male test sample on the X chromosome is 1/2 that of the normal female reference (i.e. 1 versus 2 copies of chromosome X). If the same threshold used for autosomal chromosomes was applied on X, the segmentation result will show "one copy number loss" But when corrected for gender, the segmentation analysis will no longer pick up the "copy number loss on X" because the adjusted threshold takes into account that the male sample will have 1/2 the intensity on the X chromosome compared to that of the female reference. Therefore, the automatic threshold adjustment feature allows the segmentation analysis to take into account the sex-mismatch hybridization so only copy number changes on the X chromosome that deviated from the expected normal ratio was reported. Chromosome Y was excluded in the analysis.

Concordance between aCGH profiles was measured using weighted Pearson correlation. A weighted Pearson measure was used because, in aCGH comparisons, unweighted Pearson correlation is driven by the number of ratios that differ from "normal." Since the observations in this case are the clones, one can use the spatial relationship of the clones to estimate the weights. Based on this idea, the weight of a clone was estimated by considering its deviation and that of its adjacent clone from "normal" The formula used for calculating weighted correlation coefficient (corr) is given below:

$$r_{w}=\frac{\text{cov}\;\left(x,y;w\right)}{\text{cov}\;\left(x,x,w\right)\text{cov}\;\left(y,y;w\right)},$$

where x and y are the log2 ratio values of the two samples under consideration; w is the weight vector; $w_{i}=\frac{1}{2}\left(median\left(\left|x_{j}-x_{ref}\right|\right)+median\left(\left|y_{j}-y_{ref}\right|\right)\right)$; i = 1,2,...,n-1; j = i, i + 1; n = total number of clones; and *x_ref _*and *y_ref _*are the medians of the n clones for samples x and y, respectively. Only autosomal clones were considered. Correlation coefficients falling in the intervals 0 to < 0.20, 0.20 to < 0.40, 0.40 to < 0.60, 0.60 to < 0.80 and 0.80 to 1 were said to be un-, lowly-, fairly-, moderately- and highly- correlated, respectively.

## Results

### Methods development

**Figure 1:**
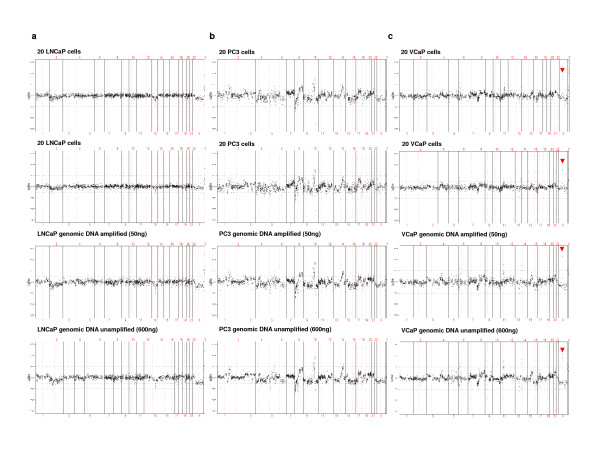
**Copy number analysis of spiked cells isolated from healthy blood via IE/FACS**. Twenty (20) isolated (A) LNCaP, (B) PC3 and (C) VCaP cells (in duplicate) and 50 ng of genomic DNA from cell culture subjected to whole genome amplification, and unamplified genomic DNA (600 ng) from cell culture (positive control) were analyzed for copy number aberrations. The log2 ratio value for each BAC clone is plotted on the y-axis. The x-axis represents the genomic position of each BAC clone on the array, with chromosome numbers indicated. Vertical solid lines indicate chromosome boundaries, and vertical red dashed line represents the centromeric region dividing each chromosome into the p- or short arm (to the left of centromere) and the q- or long arm (to the right of the centromere). Arrows indicate high-level gains on X chromosome region containing *AR *observed in VCaP cells.

For proof of principle studies using a spiked tumor cell model system, we chose LNCaP, PC3, and VCaP cell lines because they have well-characterized copy number aberrations [20]. Of note, initial tests on diluted genomic DNA and isolated spiked cells using breast cancer cell line BT474 showed that the optimal input for WGA which yielded robust aCGH results was ~20 cells (data not shown). For each prostate cell line, 1000 trypsinized cells were spiked into 10 mL of whole blood from healthy volunteers. Twenty (20) tumor cells were collected in duplicate via IE/FACS. Isolated cells were then subjected to WGA and aCGH analysis. The resulting genomic profiles demonstrated the expected known copy number aberrations (Figure 1A-C), as did aCGH profiles from amplified genomic DNA (50 ng) and non-amplified genomic DNA (600 ng) performed as a positive control. Profiles derived from 20 tumor cell-inputs revealed moderate to high correlation with the positive control (Table 2) indicating minimal dilution from contaminating normal leukocyte DNA, which would have otherwise dampened dynamic range [21]. Furthermore, consistent with previous genomic studies on VCaP cells [20], a focal amplification in the X-chromosome region containing AR was observed (Figure 1C).

**Table 2:** Correlation analysis of genomic profiles of spiked cells isolated from blood via IE/FACS.

Cell Line	Source	Input for WGA	No. of replicates	Correlation with positive control	**S.D**.
LNCaP	IE/FACS	20 cells	2	0.63	0.15
	Cell culture	50 ng	1	0.82	n.a.
PC3	IE/FACS	20 cells	2	0.88	0.03
	Cell culture	50 ng	1	0.97	n.a.
VCaP	IE/FACS	20 cells	2	0.82	0.02
	Cell culture	50 ng	1	0.89	n.a.

Isolated 20 tumor cells (in duplicate) and genomic DNA (50 ng) from cell culture were subjected to WGA and aCGH analysis and compared to positive control: unamplified genomic DNA (600 ng) from cell culture

### Enumeration, isolation and aCGH analysis of CTCs

Twenty (20) sequential CRPC patients were assayed for CTC level by CellSearch™ (see Additional file 2: Figure S2 for study schema). Fifteen (15) patients showed ≥ 5 CTCs per 7.5 mL, the cutoff used in prior clinical trials of this assay in metastatic prostate cancer [6], with mean = 12.2 and median = 3.0 CTC/mL (Table 1). Of these 15 patients who were positive for CTCs, twelve (12) were subjected to CTC isolation via IE/FACS. Three patients were excluded from further analysis because of insufficient amount of remaining blood. Nine (9) were successfully analyzed for copy number aberrations. Three of the 12 samples that met the established parameters to move forward with IE/FACS did not yield informative aCGH results (25% failure rate) due to FACS instrument error, inadequate WGA yield, or failed microarray hybridization. Two of the samples (patients #15 and #19) were processed 2 days after blood draw (Table 1). Based on this observation, we suggest implementing IE/FACS within 24 h after blood draw.

**Figure 2:**
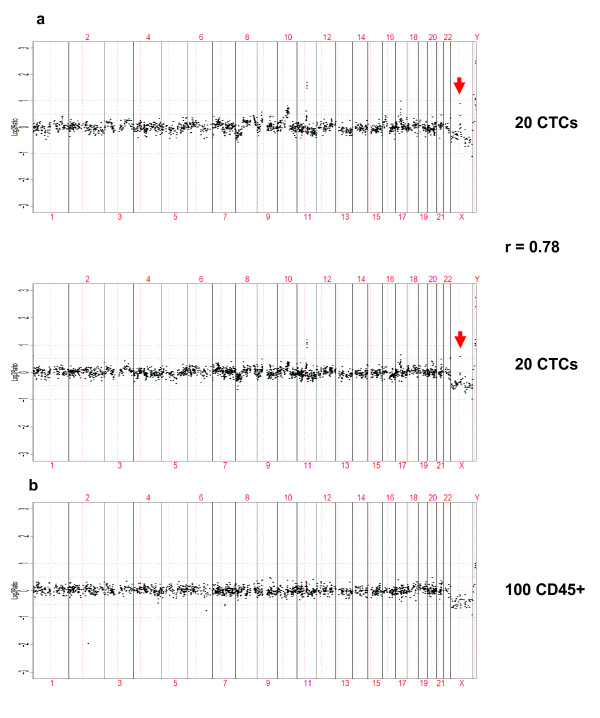
**Copy number analysis of CTCs**. Array comparative genomic hybridization analysis of (A) 20 CTCs in duplicate (B) and 100 leukocytes (CD45-positive) isolated from the same enriched blood sample from patient #9. The log2 ratio value for each BAC clone is plotted on the y-axis. The x-axis represents the genomic position of each BAC clone on the array, with chromosome numbers indicated. Vertical solid lines indicate chromosome boundaries, and vertical red dashed line represents the centromeric region dividing each chromosome into the p- or short arm (to the left of centromere) and the q- or long arm (to the right of the centromere). Arrows indicate high-level gains on X chromosome region containing *AR*.

Isolated CTCs from these CRPC patients exhibited copy number aberrations indicative of malignant origin. Due to the paucity of FACS sorted CTCs, re-analysis by FACS to confirm purity and the correct expression of biomarkers was not performed; hence, the isolation of falsely positive "EpCAM+/CD45-" cells cannot be ruled out. However, the resulting aCGH profiles showed unequivocal evidence of cancer-associated genomic alterations, and did not appear to be significantly dampened by false positive normal cells. For example, copy number analysis of replicate CTC isolates from patient #9 revealed gains including focal amplification in 11q13 (includes *CCND1*) and high-level gains in a region on chromosome X including the *AR *locus (Figure 2A). Also, replicates were moderately correlated (r = 0.78) indicating the reproducibility of the assay. As expected, these copy number aberrations were not observed in 100 leukocytes isolated from the same enriched sample (Figure 2B, Additional file 3: Figure S3C).

**Figure 3:**
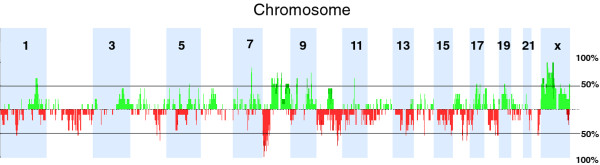
**Frequency of copy number alterations in CTCs from 9 metastatic CRPC patients**. Gains and losses are shown in green and red, respectively. Chromosome Y was not included in the analysis.

**Figure 4:**
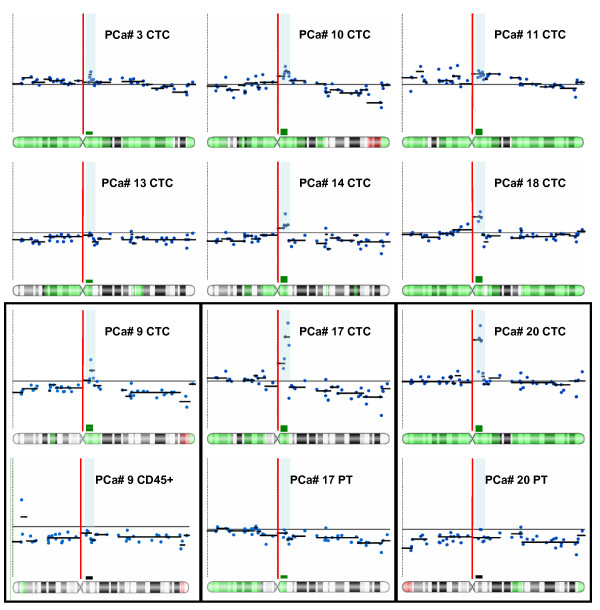
**X chromosome and *AR *locus copy number analysis in CTCs from 9 metastatic CRPC patients**. Each plot shows log2 hybridization ratio values (y-axis) at BAC clones (blue dots) distributed from the p terminus to the q terminus (x-axis). The black horizontal lines represent log2 ratio equal to 0. Red vertical lines demarcate the centromere. Broken black lines superimposed on BAC clones are the output of segmentation analysis providing high confidence copy number calls. Transparent blue bars identify the locus containing the androgen receptor gene (*AR*). At the bottom of each panel is an ideogram of chromosome X with cytoband regions showing gains (green) and losses (red). Bars on top of the ideogram (long arm) show results of copy number analysis at the *AR *locus (double green bar- high-level copy number gain; single green bar- low level copy number gain, grey bar- no copy number change). Paired samples are enclosed in black boxes: CTCs vs. CD45-positive (leukocytes) from patient #9 and CTCs vs. archival primary tumor (PT) from patients #17 and #20.

ACGH analysis of CTCs from all 9 patients revealed a wide range of copy number aberrations, including those that have been previously reported in prostate tumors ([22,23] Figure 3 and Additional file 3: Figure S3). Copy number aberrations present in > 50% of the samples include gains at 1q, 4q, 7q, 8q, 9p, 9q, 11q, 19q, Xp, and Xq and losses in 1p, 4q, 5q, 8p, 10q, 11q, 13q, 14q, 15q, and 16q (Additional file 4: Table S1). Of note, high-level gains in the region of chromosome X containing the *AR *locus were observed in 7 (78%) of the 9 cases, while low level copy number gains were observed in the remaining 2 (22%) cases (Figure 4). These results suggest that CTCs may reveal evidence for *AR *amplification, which has been associated with disease progression in CRPC [24].

### CTCs versus matched archival primary tumors

In patients #17 and #20, archival tissue previously obtained at time of primary tumor diagnosis, and prior to any cancer therapy, was available. These tumor specimens were subjected aCGH analysis and compared to the corresponding CTC samples.

**Figure 5:**
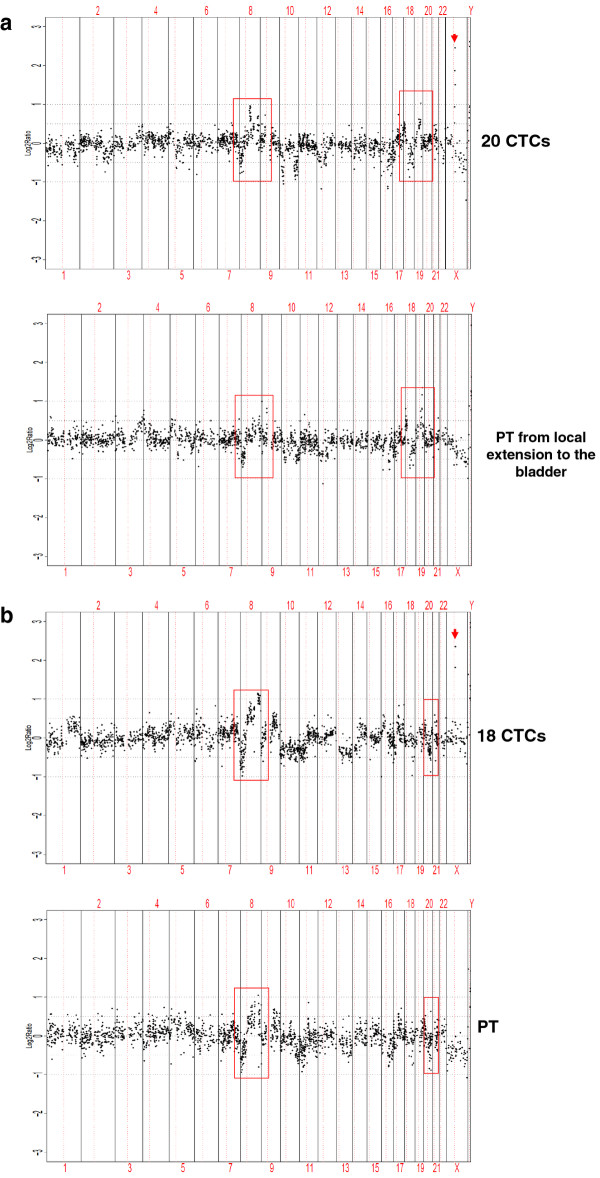
**Copy number analysis of CTCs versus matched archival tumor in two patients**. Array CGH analysis of (A) 20 CTCs from patient #17 and a corresponding archival primary tumor from local extension to the bladder obtained 1 year and 3 months prior to CTC analysis; and (B) 18 CTCs from patient #20 and a matched primary tumor obtained 5 years and 1 month prior to CTC analysis. Arrows indicate high-level gains on X chromosome region containing *AR *in CTCs but not observed in archival primary tumors. Chromosome regions in red boxes show genomic aberrations common to both paired samples (Also see Additional file 3: Figure S3).

Patient #17 is a 48-year old CRPC patient initially diagnosed with prostatic adenocarcinoma with Gleason score 9 (4 + 5), and with local extension to the urinary bladder. The aCGH analysis of his CTCs revealed genomic aberrations including high-level gains at 18p, 19q and the *AR *locus of chromosome X; and losses at 8p and 18q (Figure 5A, Additional file 3: Figure S3H). The archival primary tumor specimen involving the bladder from this same patient, which dated to 1 year and 3 months prior to CTC analysis, was obtained for aCGH analysis as well. Only tumor from the bladder yielded informative aCGH profile (Additional file 3: Figure S3I). Comparison of the respective genomic profiles revealed shared copy number aberrations indicative of common lineage, including losses in 8p and 18q as well as gains in 8q, 18p and 19q (Additional file 3: Figure S3A). Conversely, new copy number aberrations were observed in CTCs but not in the primary tumor, including copy number gain at the *AR *locus (Figure 4).

Patient #20 is a 67-year old CRPC patient initially diagnosed with prostatic adenocarcinoma with Gleason score 8 (4 + 4). ACGH analysis of his CTCs revealed genomic aberrations including high-level gains in 8q and in a region on chromosome X containing *AR*, as well as loss of 13q (Figure 5B; Additional file 3: Figure S3K). ACGH analysis of the corresponding primary tumor (Additional file 3: Figure S3L) obtained via core biopsy 5 years and 1 month prior to CTC analysis revealed similarities in copy number alterations when compared with the CTCs, including losses in 8p, 10q, and 11p and gains in 8q and 9q (Additional file 5: Figure S4B). In addition to these conserved genomic aberrations, new copy number changes were evident, particularly the *AR *region gain shown in CTCs but not in the primary tumor (Figure 4).

## Discussion

In this pilot study, we assessed the feasibility of a new approach to isolate and genomically profile CTCs in patients with CRPC. Molecular profiling of CTCs revealed genomic aberrations that have been previously described in prostate cancer including gains in 8q, 11q13 and losses in 8p [22,23].

Our approach involving immunomagnetic enrichment, FACS sorting and aCGH analysis is novel, and this report confirms the feasibility of this approach for genomic analysis of isolated CTCs. Immunomagnetic enrichment alone provides a cell mixture which is predominantly hematopoietic, although further analysis may be performed on the admixture of cells [25-27]. In a notable prior report, Racila et al. described immunomagnetic enrichment followed by flow cytometry for enumeration of CTCs, as well as enrichment followed by cytospin for immunostaining [28]. Our approach enables full FACS-based isolation of CTCs for molecular profiling.

Interestingly, we observed copy number gains in the AR region of chromosome X of CTCs, including high-level gains in 78% of the samples which were successfully profiled. AR amplification is not a common event in primary prostate cancer, but has been implicated in hormone resistance in CRPC [24,29]. In the two patients with matching archival tumor and subsequent CTC specimens, high-level gain in the AR region was observed in the CRPC CTCs but not in the archival tumors. The gain in AR copy number between tumor tissue obtained at initial diagnosis and CTCs subsequently obtained during the CRPC phase may reflect selective pressures towards amplification of the AR in response to androgen deprivation therapy. Although our approach for whole genome amplification and aCGH of isolated CTCs is not designed to be a clinical test, it is possible that the observations here, such as focal amplification in the X chromosome (AR) in CTCs, could serve as the basis for new CTC-based clinical applications. Finally, we performed manual microdissection of archival samples, which can potentially include adjacent benign tissue. Moreover, since prostate cancer is multifocal [30], the use of laser microdissection to isolate different clones present in the primary tumor will further refine our understanding of the clonal relationships of CTCs and their primary tumor focus of origin.

A major limitation of this study is that it was designed as a pilot feasibility study with a small sample size. Another potential limitation of this approach is that it was only focused on EpCAM-positive CTCs. EpCAM-based selection methods, such as IE/FACS and CellSearch™, may miss CTCs expressing low levels of EpCAM. The clinical significance of EpCAM-negative CTCs has yet to be determined, but it has been hypothesized that this may include cells undergoing epithelial-mesenchymal transition [31].

Molecular studies using specific FISH probes on CTCs from advanced prostate cancer patients have noted gains in *AR *and *MYC*, losses in *PTEN*, and evidence for *ERG *gene rearrangement [32,33]. In addition, Stott et al [34] also reported *TMPRSS2-ERG *fusion in CTCs via RT-PCR. Genome-wide copy number microarray analysis has been performed on disseminated tumor cells (DTCs) obtained from bone marrow aspirates performed on prostate cancer patients [35,36]. Such efforts have been facilitated by the higher numbers of tumor cells in the bone marrow compared to blood [37]. For example, Holcomb et al [35] demonstrated the feasibility of aCGH analysis of pools of 10-20 DTCs from patients with organ-confined and metastatic disease and found more aberrations in DTCs in the latter. They also showed shared genomic aberrations between DTCs and their matched primary tumors. Recently, Paris and colleagues [38] reported on copy number analysis of CTCs from CRPC patients obtained via a cell adherence based enrichment method.

## Conclusions

In summary, our pilot study demonstrates that CTCs can be feasibly isolated away from hematopoietic cells via IE/FACS and profiled for genome-wide copy number aberrations. The resulting copy number analyses provide confirmation of the malignant nature of the EpCAM-positive cells in the blood. Most importantly, this approach may be used to explore genomic events associated with cancer progression, and may facilitate CTC-based biomarker discovery.

## Abbreviations

aCGH: array comparative genomic hybridization; AR: androgen receptor; CRPC: castration resistant prostate cancer; CTCs: circulating tumor cells; IE/FACS: immunomagnetic enrichment/fluorescence activated cell sorting; PT: primary tumor; WGA: whole genome amplification.

## Competing interests

J.W. Park: Research Grant (Veridex and BD Biosciences); Honoraria from Speakers Bureau (Veridex).

## Authors' contributions

MJMM and DP optimized WGA and aCGH experiments; JHS performed cell sorting; MJMM and EVS performed WGA and aCGH experiments; CJR collected samples and patient data; JS performed pathology review of primary tumors; MJMM, CC and EVS analyzed data; MJMM, CJR, JS and JWP wrote the manuscript. All authors discussed the results and implications and commented on the manuscript at all stages.

## Pre-publication history

The pre-publication history for this paper can be accessed here:

http://www.biomedcentral.com/1471-2407/12/78/prepub

## Supplementary Material

Additional file 1 **Figure S1 Fluorescence activated cell sorting (FACS) analysis of two representative clinical blood samples positive for CTCs**. Samples after immunomagnetic enrichment (enriched for tumor cells) were subjected to fluorescence activated cells sorting (FACS) to isolate for CTCs. Tumor cells were stained with EpCAM (EBA-1) mAb conjugated to phycoerythrin (EpCAM-PE), a nucleic acid dye and a leukocyte-specific CD45 (2D1) mAb conjugated to peridinin-chlorophyll-protein-Cy5.5 (CD45-PerCPCy5.5). Forward and side scatters (top left panel) were used for preliminary identification of cells (P1 gate) and to exclude debris. P2 gate (top right panel) was used to select for nucleated (a nucleic acid dye+) cells while gates in the lower panels were used to select for EpCAM + and CD45- (P3 gate) and EpCAM + and nucleated (a nucleic acid dye+) cells (P4 gate). CTCs must be present within gates P1 to P4, and were defined as EpCAM+, CD45-, and nucleated (a nucleic acid dye+). P5 gate (lower left panel) was used to select for CD45+, EpCAM-, and nucleated (a nucleic acid dye+) cells when sorting for leukocytes. Forward scatter (FSC) vs side scatter (SSC) plots are on a linear scale while SSC vs nucleic acid dye plots are on a semi-logarithmic scale. EpCAM-PE vs CD45-PerCP-Cy5.5 and EpCAM-PE vs nucleic acid dye are log-log plots. Click here for file

Additional file 2 **Figure S2 Study schema**. Flow diagram for enumeration, immunomagnetic enrichment and fluorescence activated cell sorting (IE/FACS), whole genome amplification (WGA) and array comparative genomic hybridization (aCGH) analysis of circulating tumor cells (CTCs) in castration resistant prostate cancer. Click here for file

Additional file 3 **Figure S3 (A-L). Copy number analysis of CTCs (N = 9), archival primary tumor (N = 2) and leukocytes (N = 1)**. For each case, the top panel shows genomic profiles and the lower panel (karyograms) shows results from segmentation analysis providing high confidence copy number calls in each sample: low level gains (green bar) and losses (red bar), high-level gains (double green bars) and homozygous deletion (double red bars). Click here for file

Additional file 4 **Table S1 Recurrent aberrations (> 50%) in circulating tumor cells isolated from 9 castration resistant prostate cancer patients**. Genes and microRNA are listed for each region. Click here for file

Additional file 5 **Figure S4 Copy number analysis of CTCs versus matched archival primary tumor in two patients**. (A) 20 CTCs from patient #17 and a corresponding archival primary tumor from local extension to the bladder obtained 1 year and 3 months prior to CTC analysis; and (B) 18 CTCs from patient #20 and a matched primary tumor obtained 5 years and 1 month prior to CTC analysis. For each case, the top panel shows frequency of genomic aberrations [gains (green) and losses (red)] between the paired samples such that chromosomal aberrations with frequency equal to 100% have been gained and lost in both CTCs and archival primary tumor. The lower panel shows output of segmentation analysis providing high confidence copy number calls for each sample. Click here for file
