# Supplementary figures and images for: Isolation and genomic analysis of circulating tumor cells from castration resistant metastatic prostate cancer
Source: BMC Cancer. 2012 Feb 28;12:78. doi: 10.1186/1471-2407-12-78 (PMC3395839; doi:10.1186/1471-2407-12-78)

Supplementary Figure 1

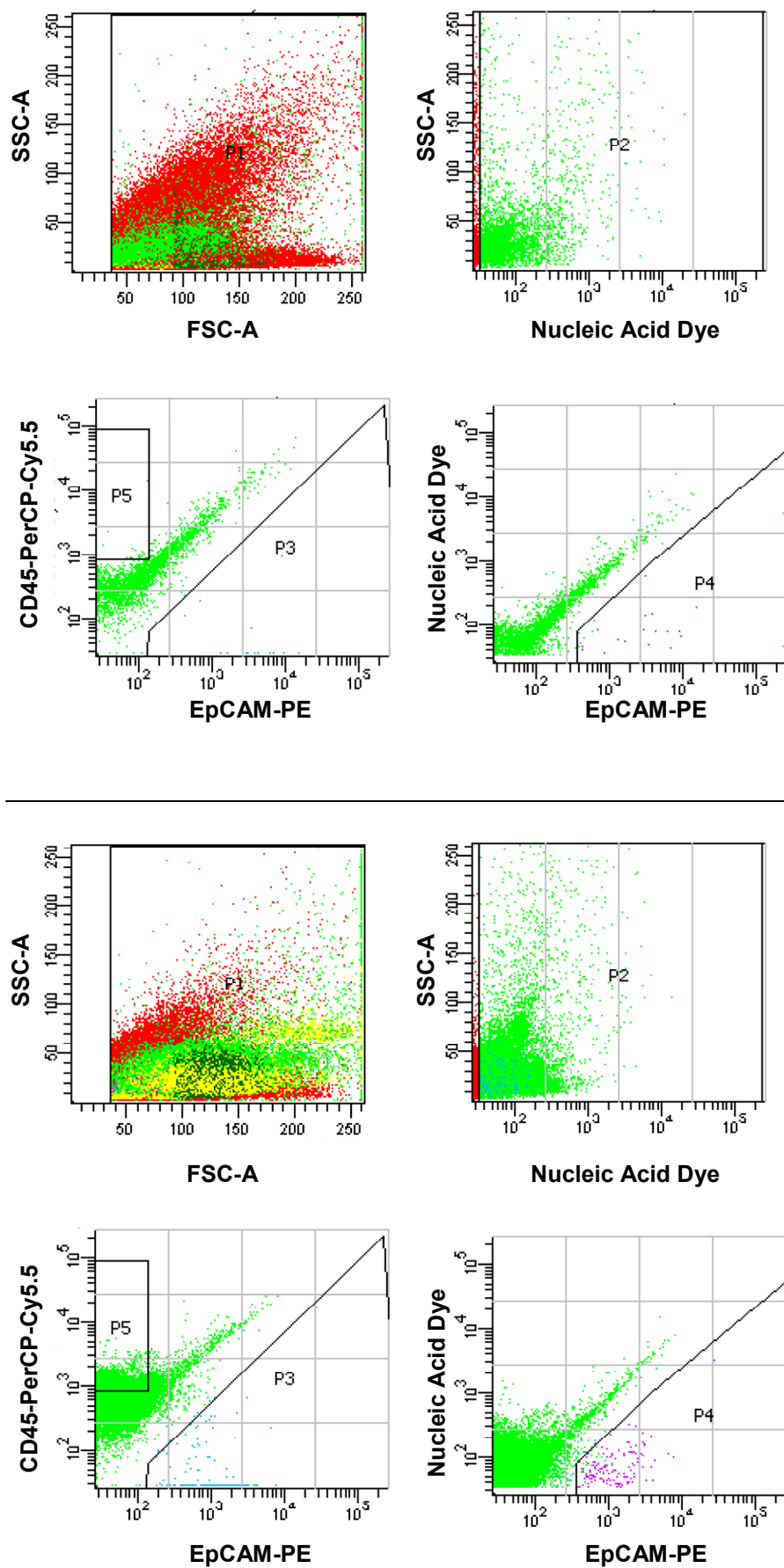

Supplement: Additional file 1 — Figure S1 Fluorescence activated cell sorting (FACS) analysis of two representative clinical blood samples positive for CTCs. Samples after immunomagnetic enrichment (enriched for tumor cells) were subjected to fluorescence activated cells sorting (FACS) to isolate for CTCs. Tumor cells were stained with EpCAM (EBA-1) mAb conjugated to phycoerythrin (EpCAM-PE), a nucleic acid dye and a leukocyte-specific CD45 (2D1) mAb conjugated to peridinin-chlorophyll-protein-Cy5.5 (CD45-PerCPCy5.5). Forward and side scatters (top left panel) were used for preliminary identification of cells (P1 gate) and to exclude debris. P2 gate (top right panel) was used to select for nucleated (a nucleic acid dye+) cells while gates in the lower panels were used to select for EpCAM + and CD45- (P3 gate) and EpCAM + and nucleated (a nucleic acid dye+) cells (P4 gate). CTCs must be present within gates P1 to P4, and were defined as EpCAM+, CD45-, and nucleated (a nucleic acid dye+). P5 gate (lower left panel) was used to select for CD45+, EpCAM-, and nucleated (a nucleic acid dye+) cells when sorting for leukocytes. Forward scatter (FSC) vs side scatter (SSC) plots are on a linear scale while SSC vs nucleic acid dye plots are on a semi-logarithmic scale. EpCAM-PE vs CD45-PerCP-Cy5.5 and EpCAM-PE vs nucleic acid dye are log-log plots. [file 1471-2407-12-78-S1.PDF]

## Supplementary Figure 2

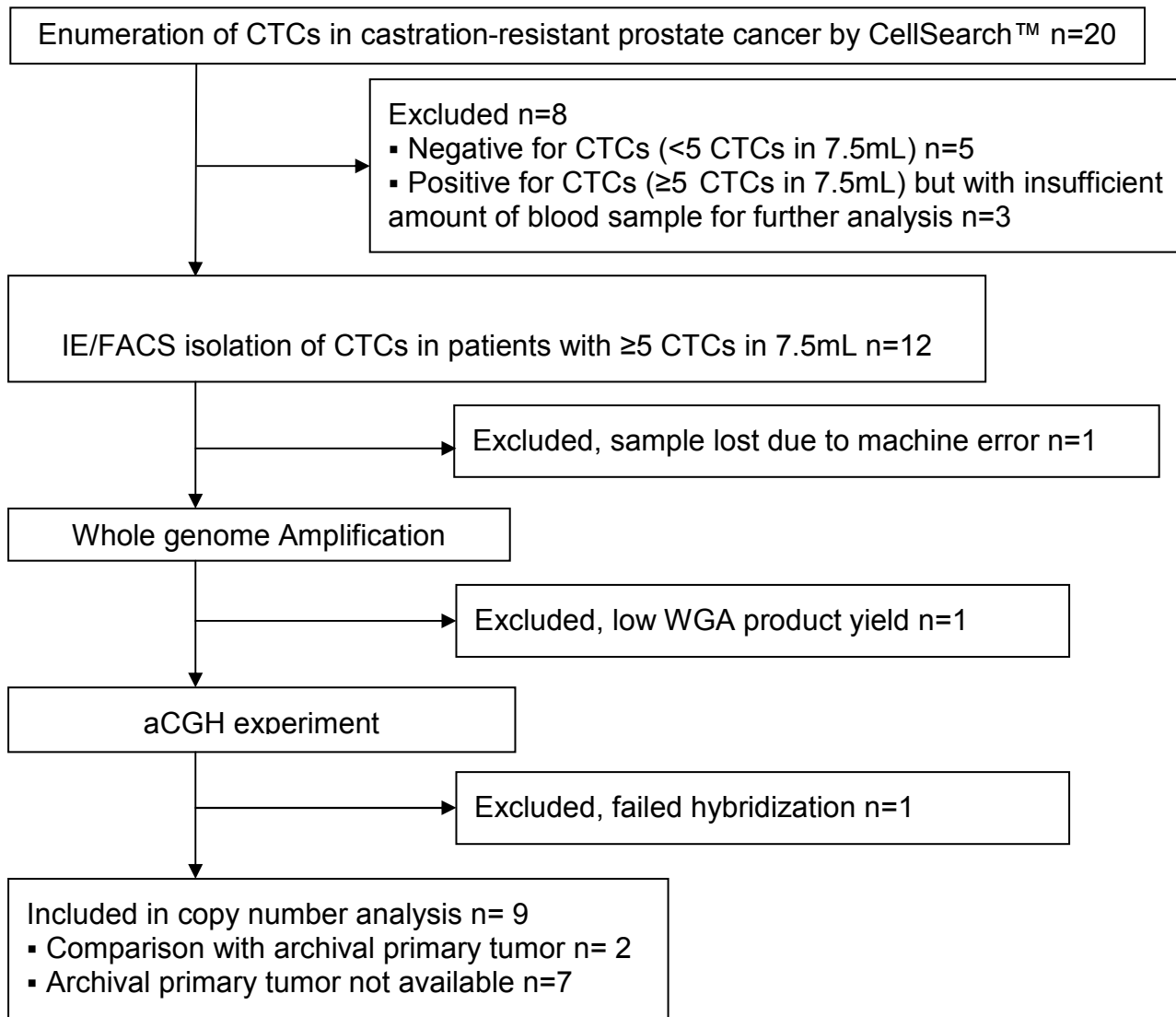

Supplement: Additional file 2 — Figure S2 Study schema. Flow diagram for enumeration, immunomagnetic enrichment and fluorescence activated cell sorting (IE/FACS), whole genome amplification (WGA) and array comparative genomic hybridization (aCGH) analysis of circulating tumor cells (CTCs) in castration resistant prostate cancer. [file 1471-2407-12-78-S2.PDF]

Supplementary Figure 4

A) PCa #17

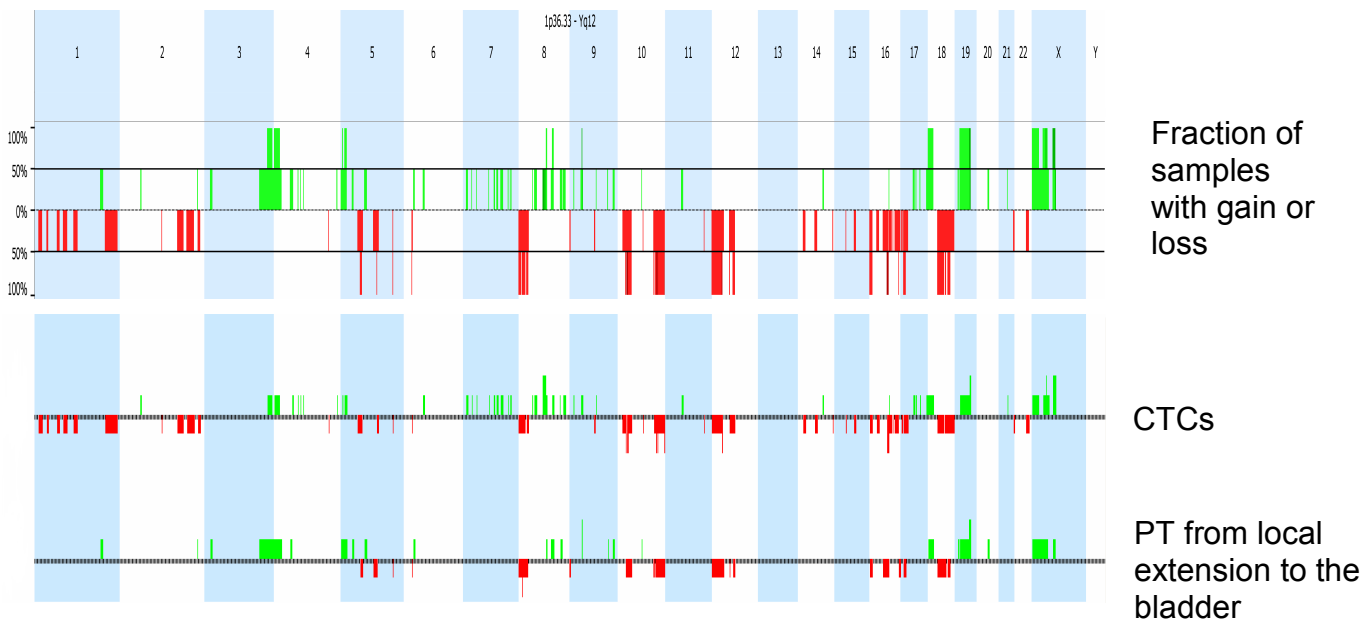

B) PCa #20

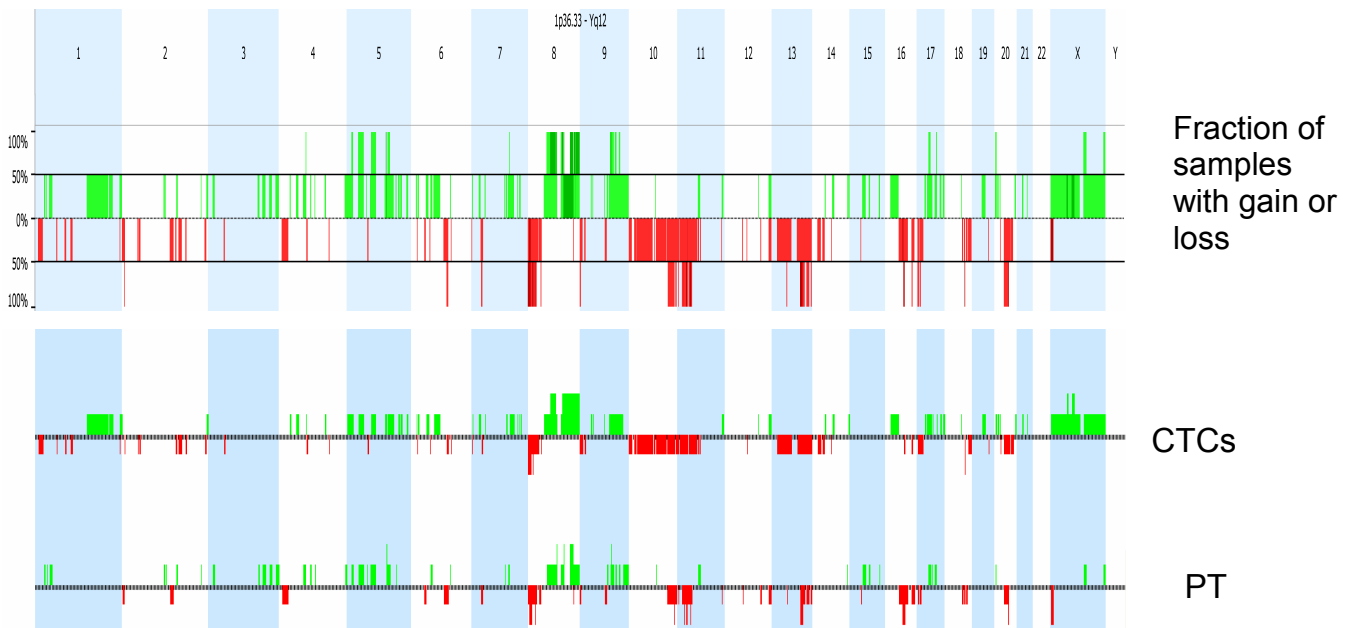

Supplement: Additional file 5 — Figure S4 Copy number analysis of CTCs versus matched archival primary tumor in two patients. (A) 20 CTCs from patient #17 and a corresponding archival primary tumor from local extension to the bladder obtained 1 year and 3 months prior to CTC analysis; and (B) 18 CTCs from patient #20 and a matched primary tumor obtained 5 years and 1 month prior to CTC analysis. For each case, the top panel shows frequency of genomic aberrations [gains (green) and losses (red)] between the paired samples such that chromosomal aberrations with frequency equal to 100% have been gained and lost in both CTCs and archival primary tumor. The lower panel shows output of segmentation analysis providing high confidence copy number calls for each sample. [file 1471-2407-12-78-S5.PDF]
