# Supplementary material for: Isolation and genomic analysis of circulating tumor cells from castration resistant metastatic prostate cancer
Source: BMC Cancer. 2012 Feb 28;12:78. doi: 10.1186/1471-2407-12-78 (PMC3395839; doi:10.1186/1471-2407-12-78)

Supplementary Figure 3

A)

PCa #3 50CTCs

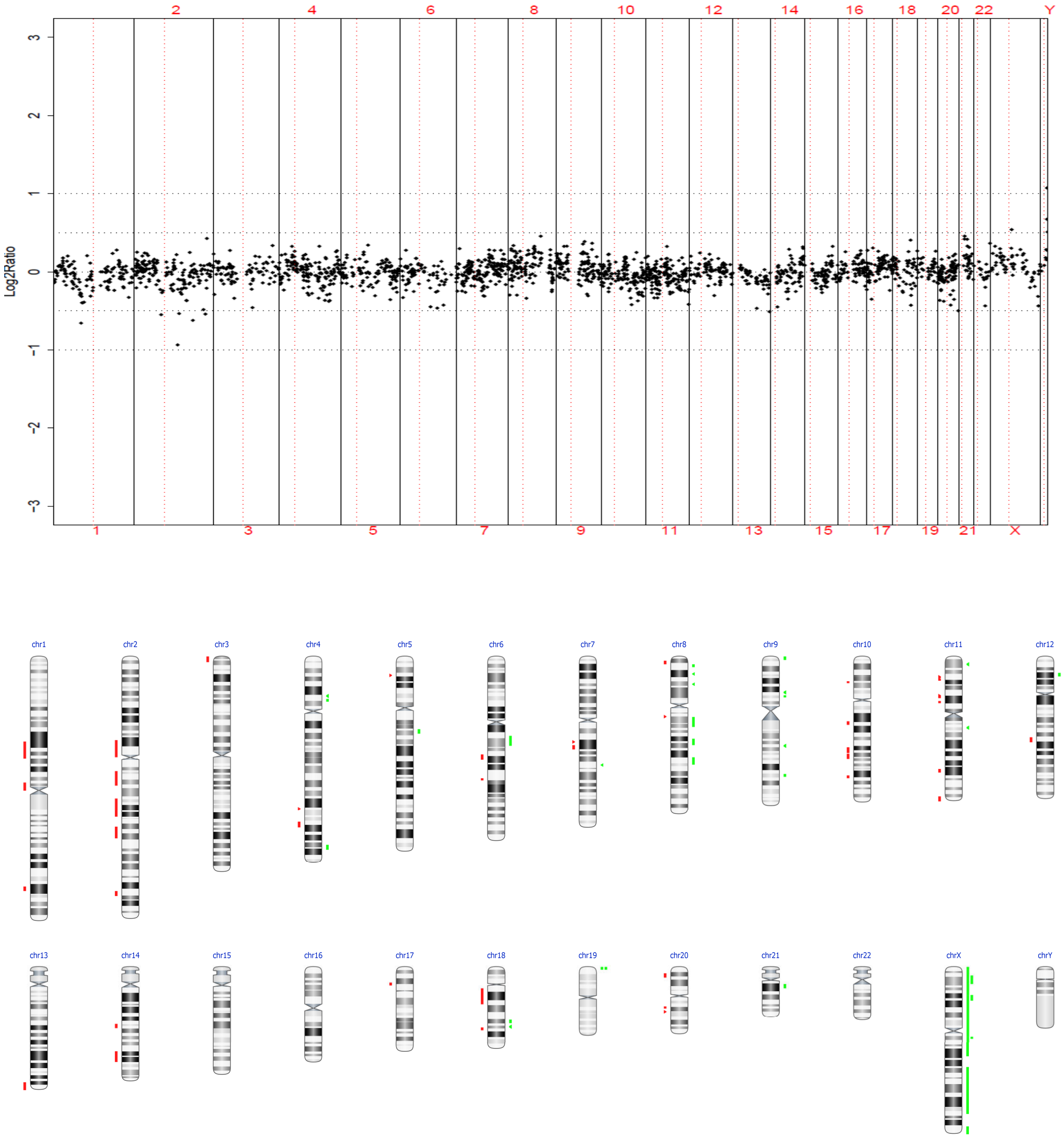

B)

PCa #9 20CTCs

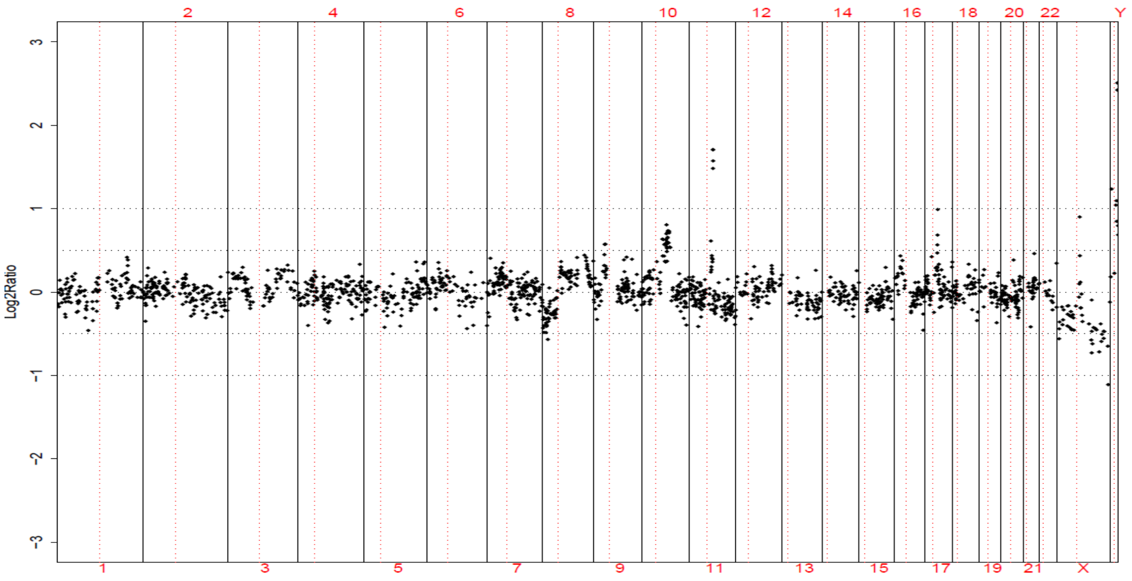

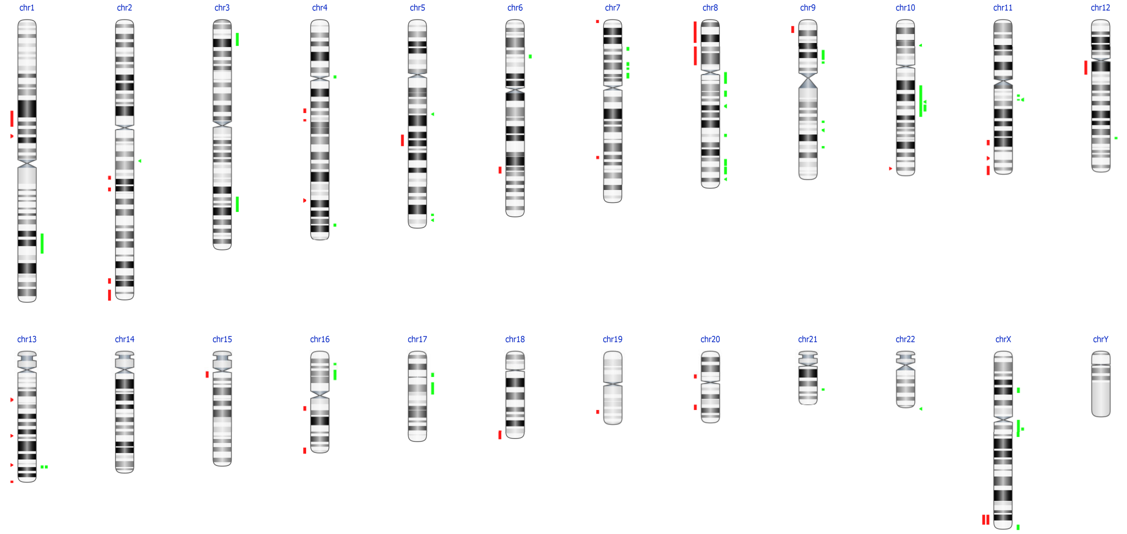

C)

PCa #9 100CD45+

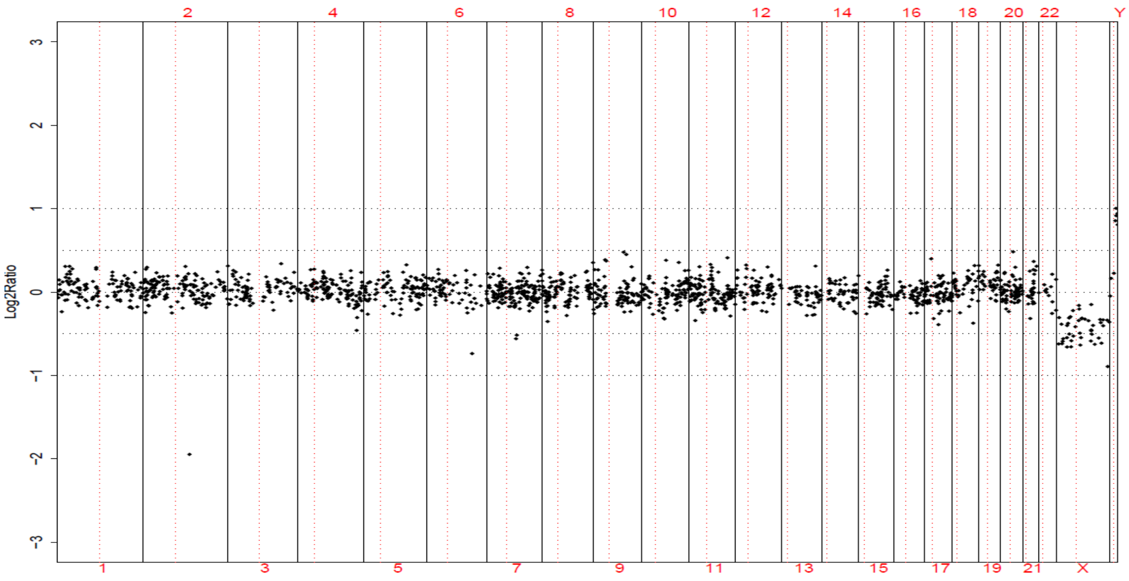

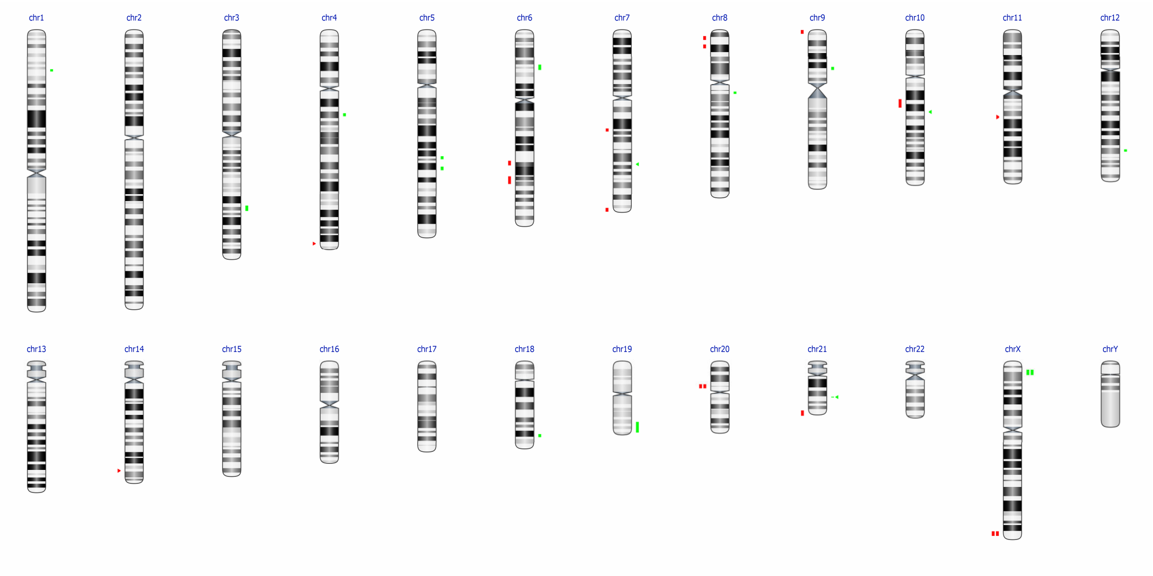

D)

PCa #10 20CTCs

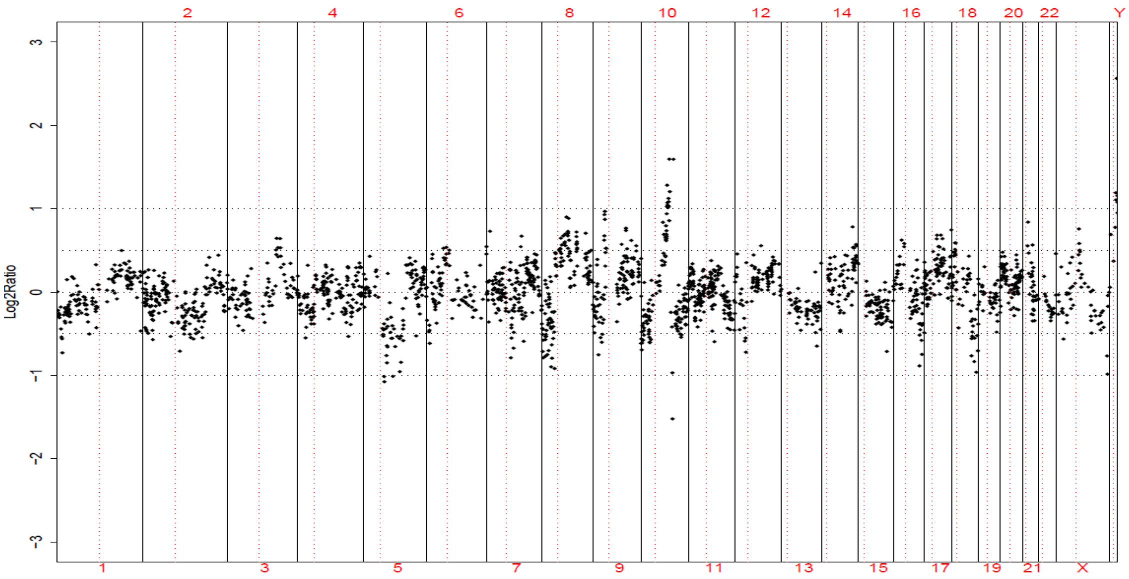

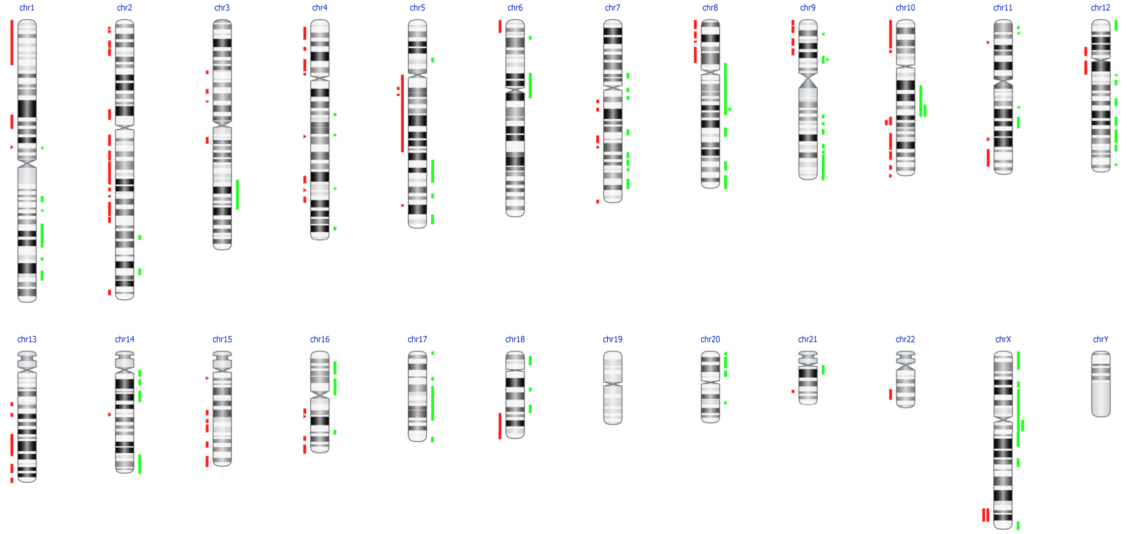

E)

PCa #11 20CTCs

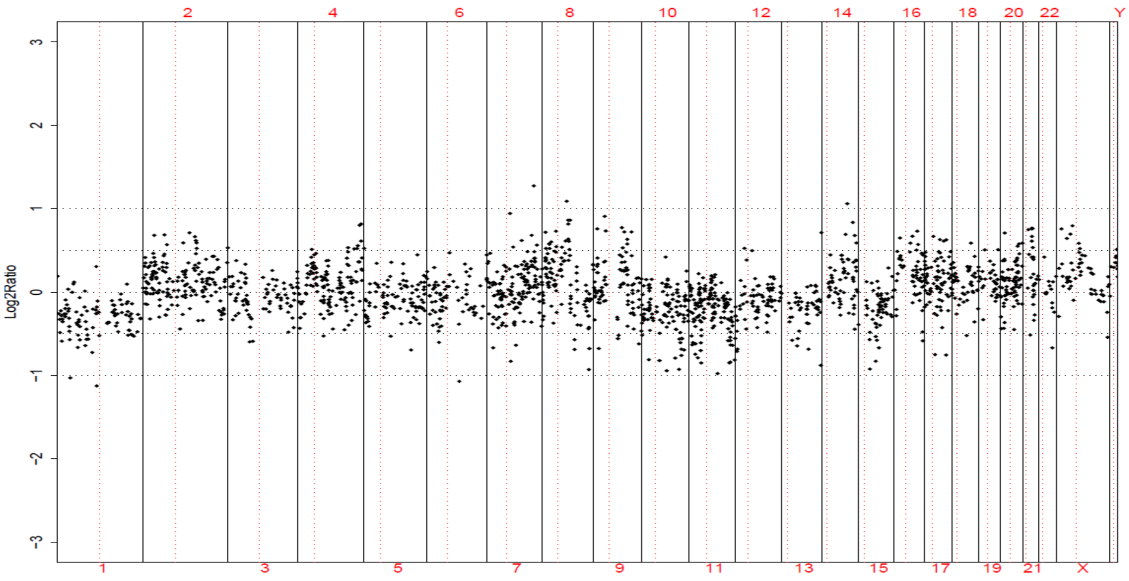

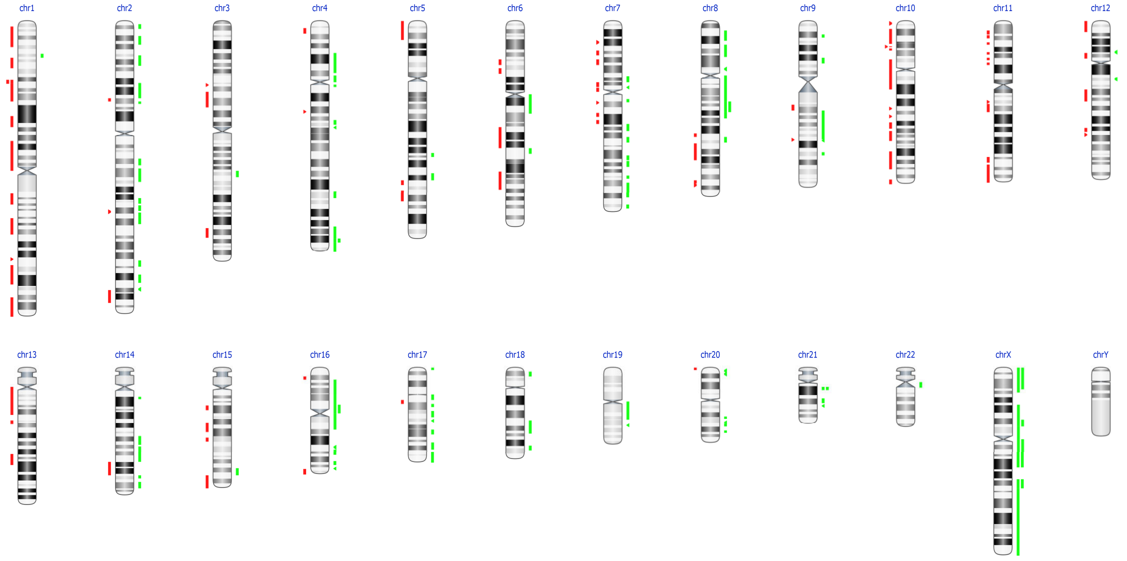

F)

PCa #13 20CTCs

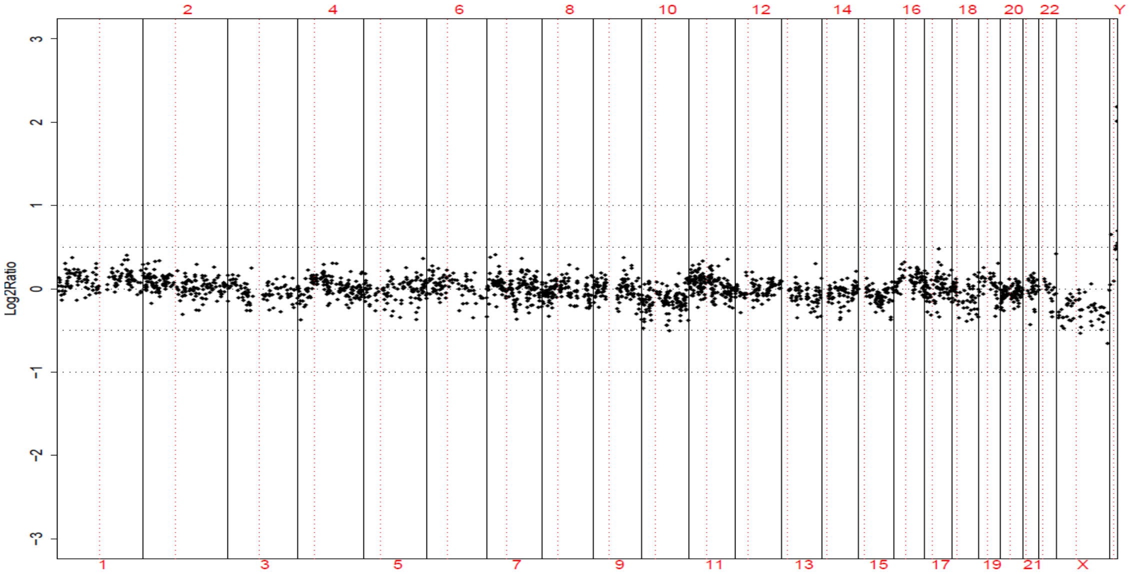

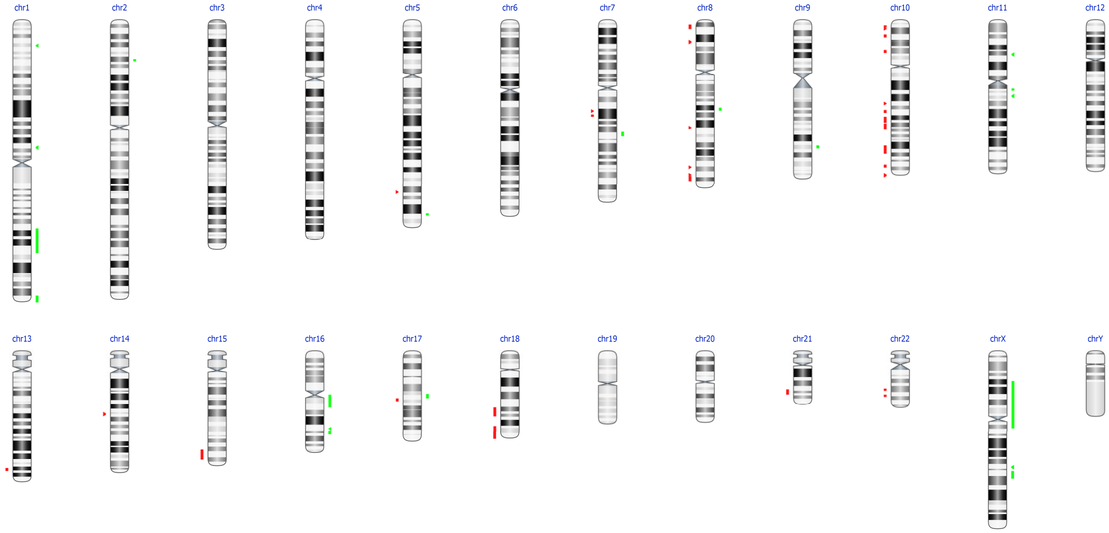

G)

PCa #14 20CTCs

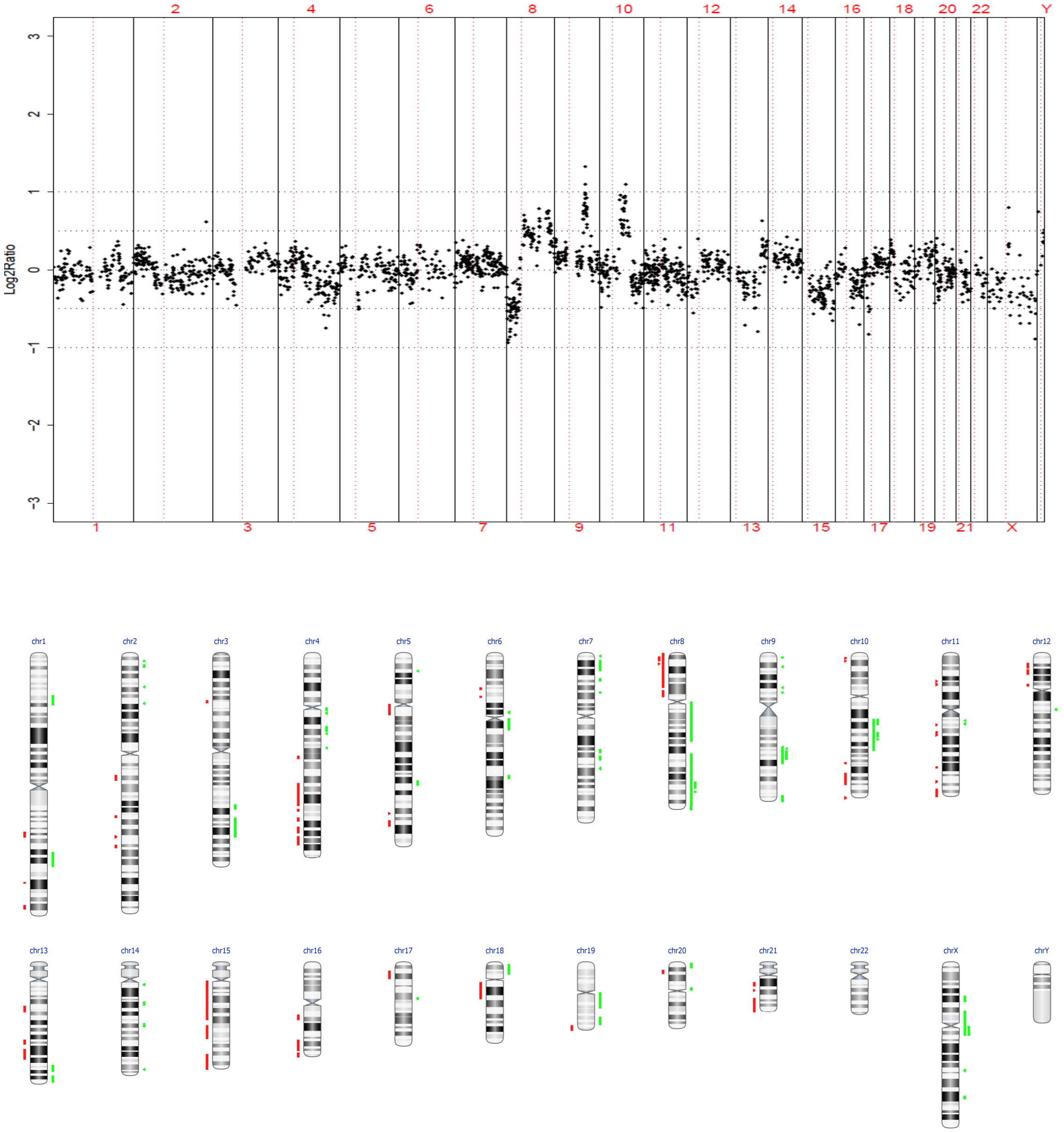

H)

PCa #17 20CTCs

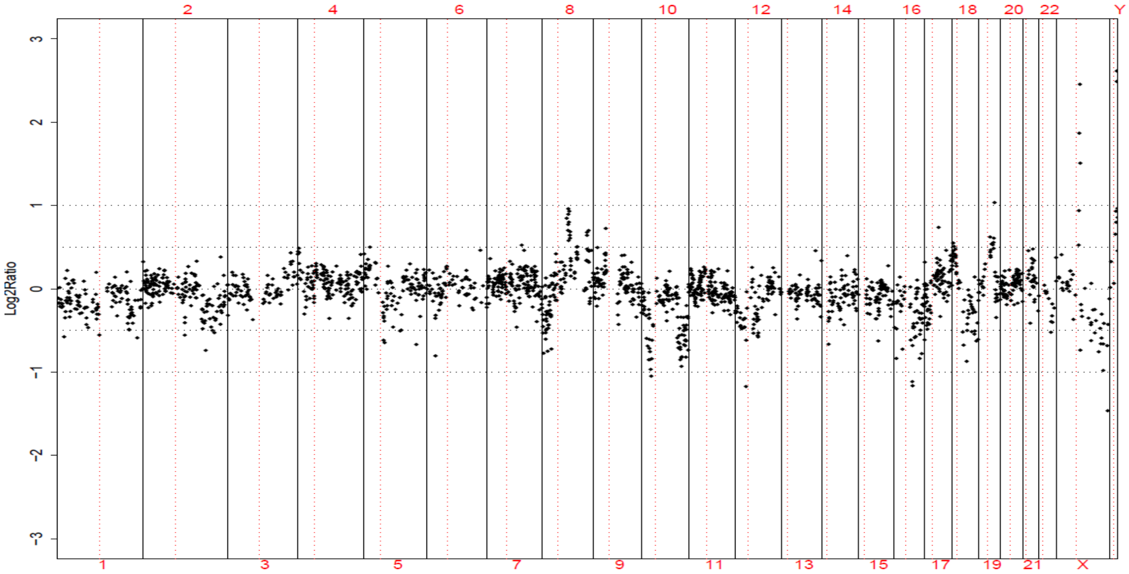

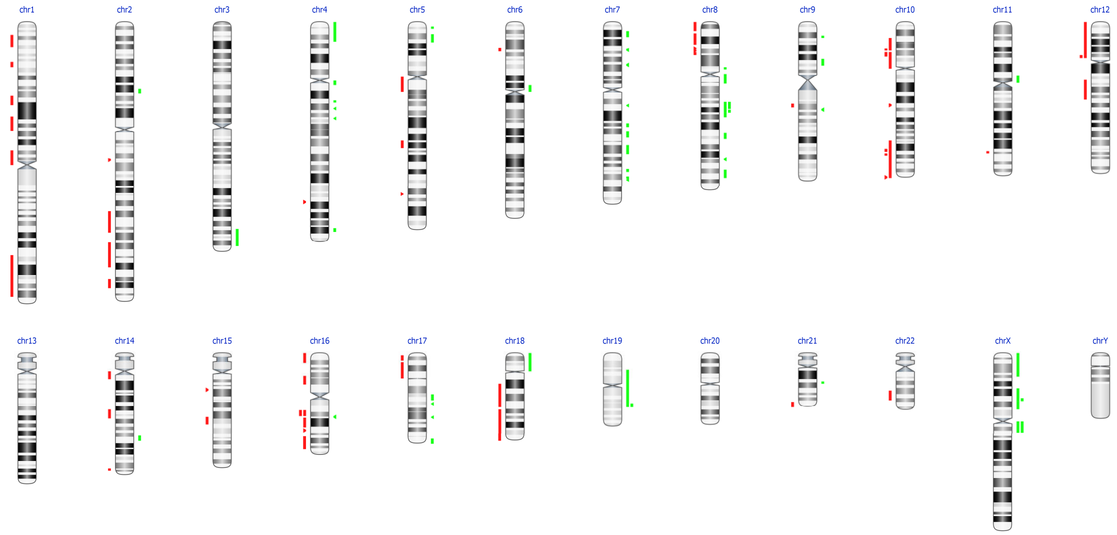

I)

PCa #17 Primary tumor from local extension to the bladder

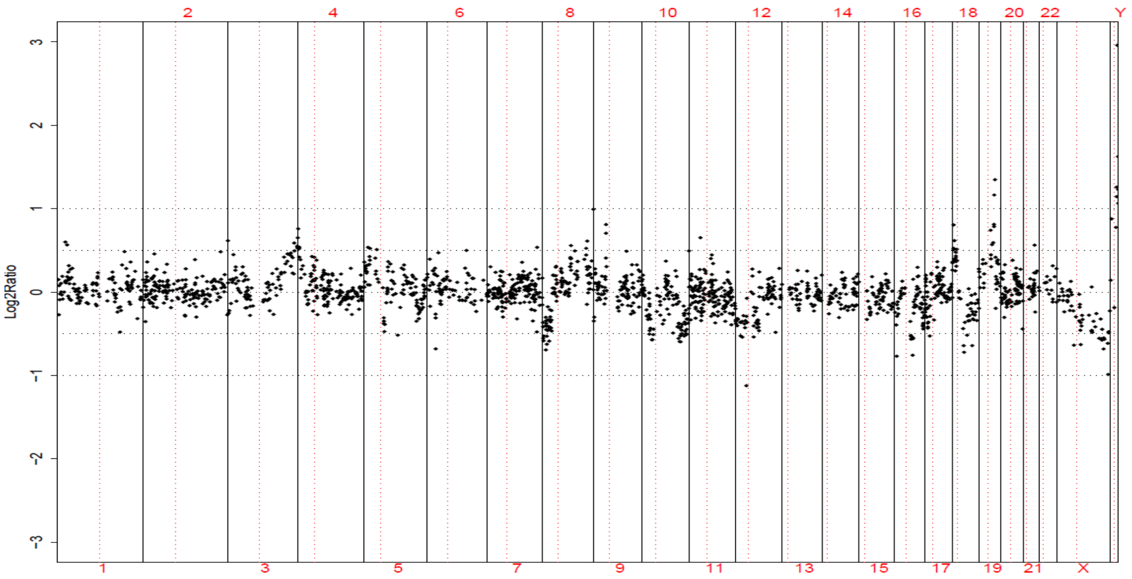

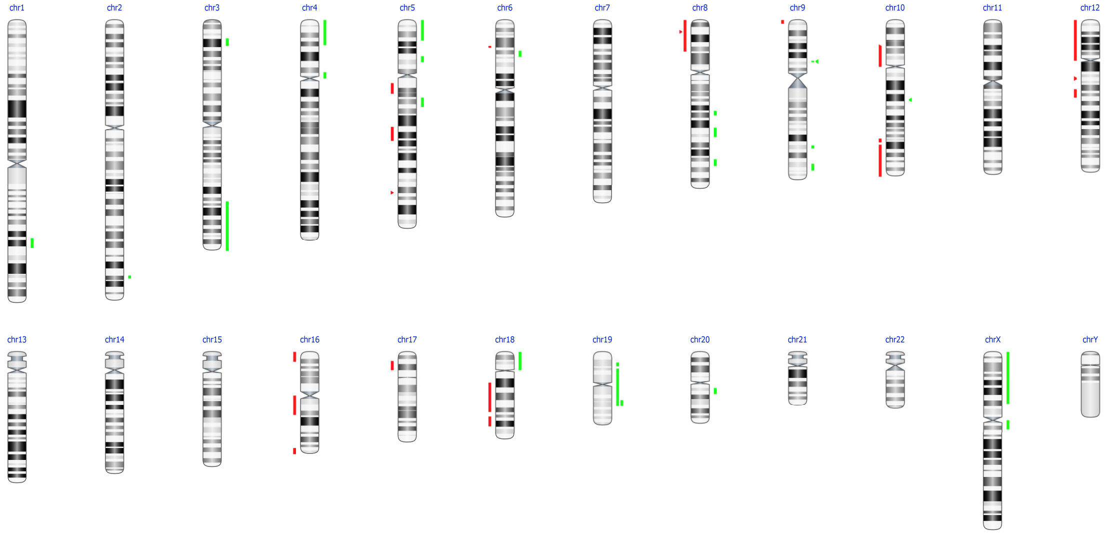

J)

PCa #18 20CTCs

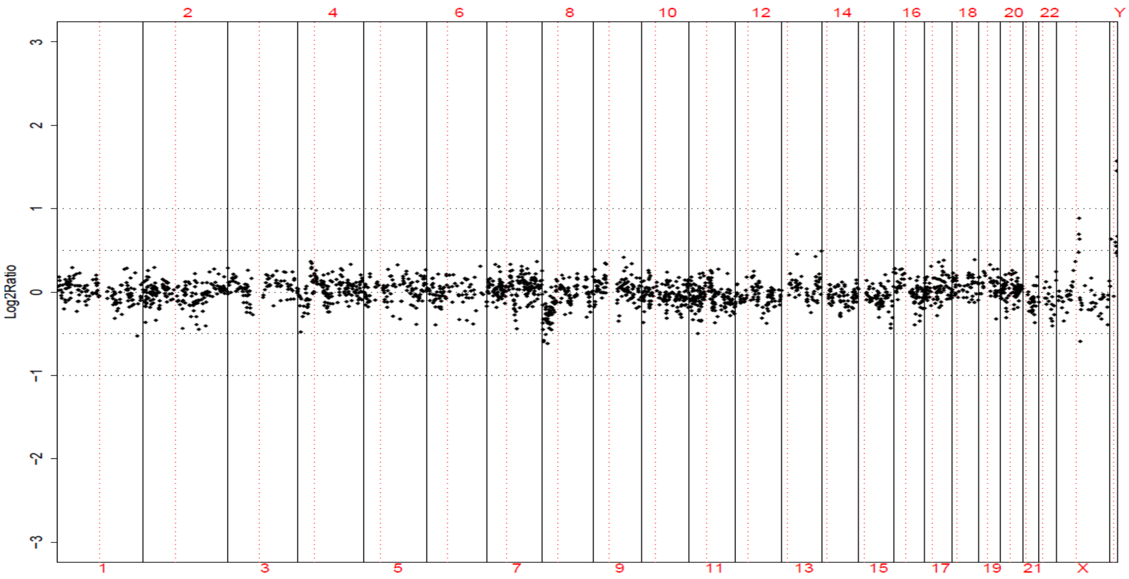

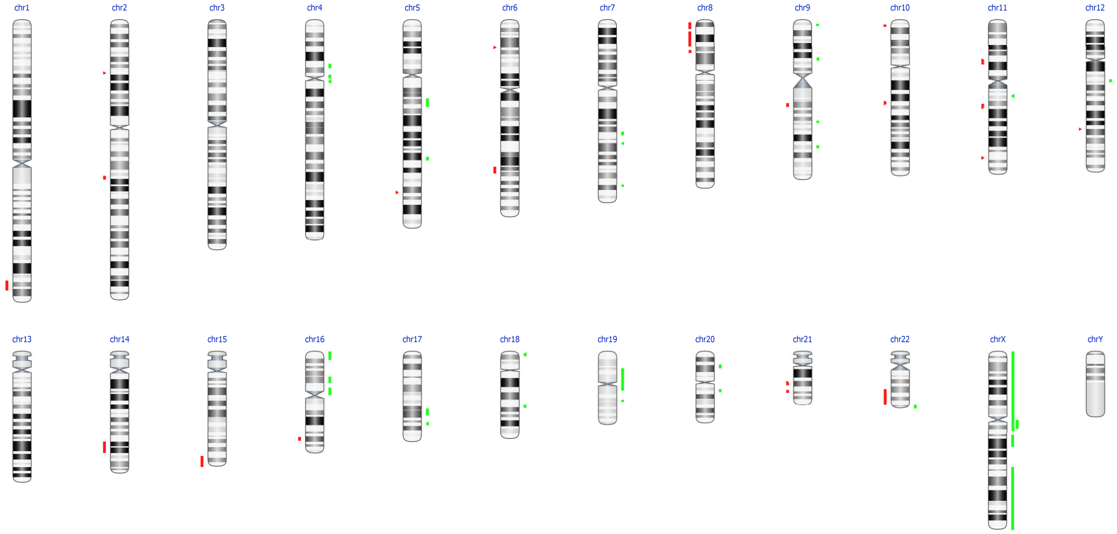

K)

PCa #20 18CTCs

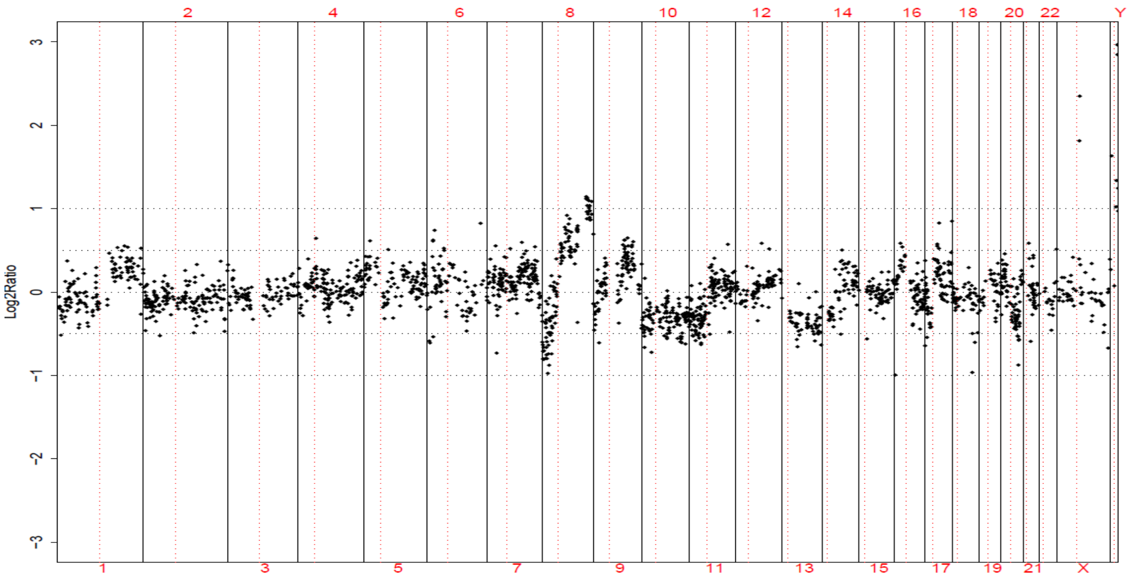

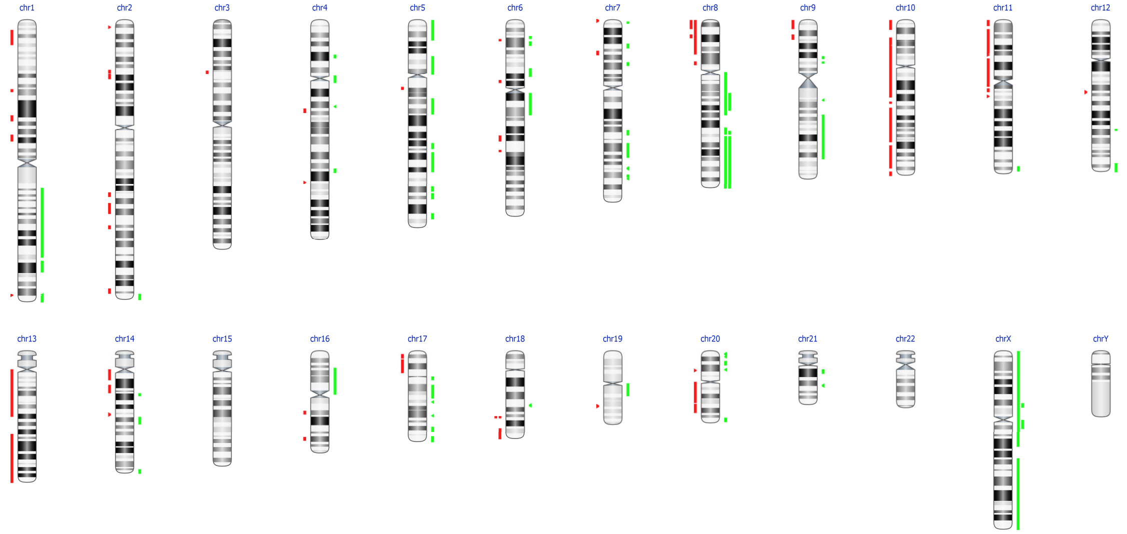

L)

PCa #20 Primary Tumor

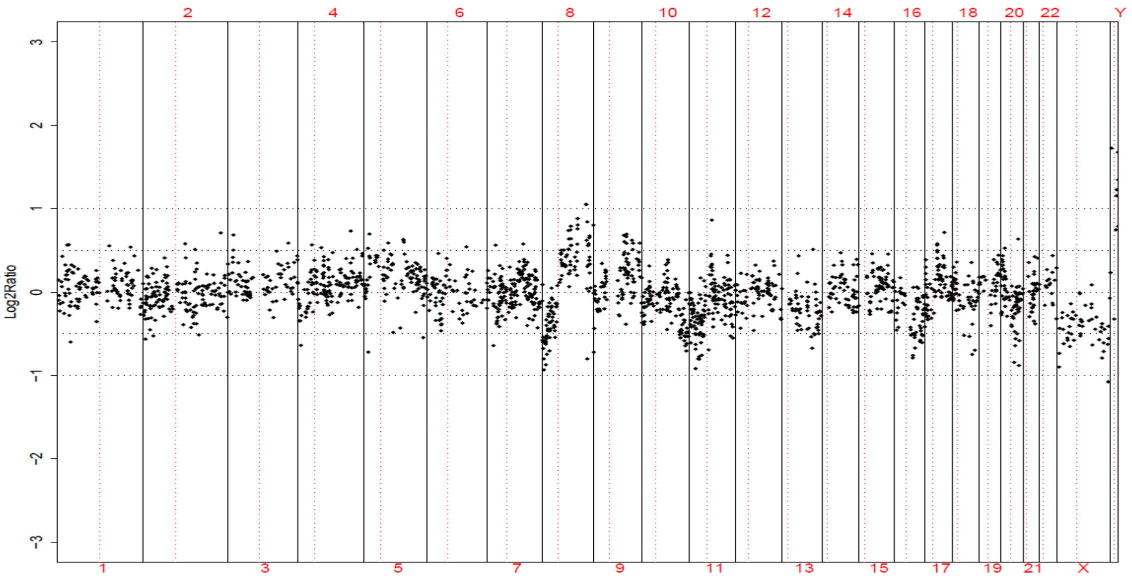

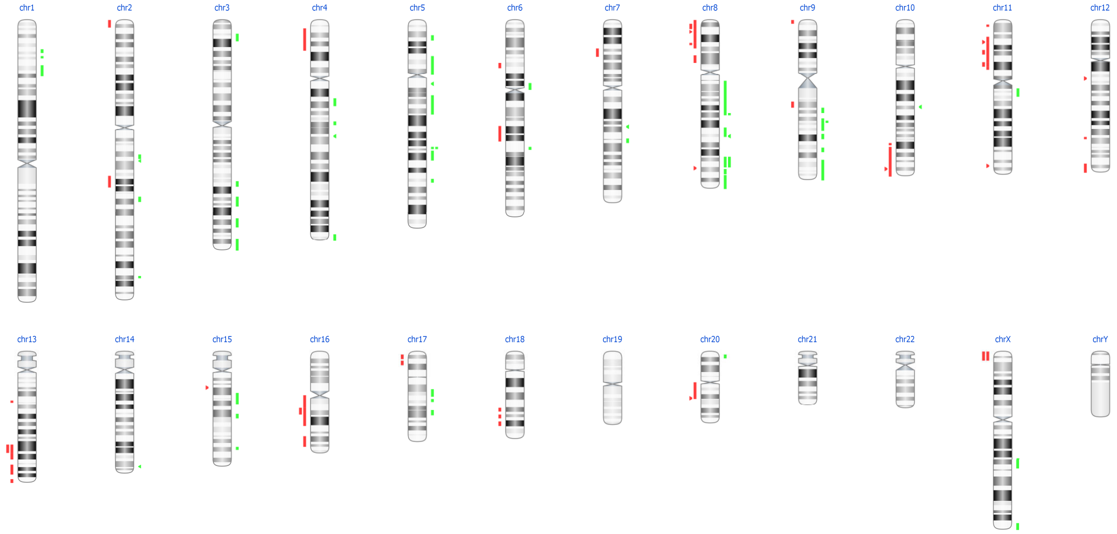

Supplement: Additional file 3 — Figure S3 (A-L). Copy number analysis of CTCs (N = 9), archival primary tumor (N = 2) and leukocytes (N = 1). For each case, the top panel shows genomic profiles and the lower panel (karyograms) shows results from segmentation analysis providing high confidence copy number calls in each sample: low level gains (green bar) and losses (red bar), high-level gains (double green bars) and homozygous deletion (double red bars). [file 1471-2407-12-78-S3.PDF]
